# Current Status and Future Opportunities in Modeling Clinical Characteristics of Multiple Sclerosis

**DOI:** 10.3389/fneur.2022.884089

**Published:** 2022-05-27

**Authors:** Joshua Liu, Erin Kelly, Bibiana Bielekova

**Affiliations:** Neuroimmunological Diseases Section (NDS), National Institute of Allergy and Infectious Diseases (NIAID) of the National Institutes of Health (NIH), Bethesda, MD, United States

**Keywords:** multiple sclerosis (MS), predictive models, machine learning, clinical outcomes, MS disability, MS severity, technical quality, reproducibility

## Abstract

Development of effective treatments requires understanding of disease mechanisms. For diseases of the central nervous system (CNS), such as multiple sclerosis (MS), human pathology studies and animal models tend to identify candidate disease mechanisms. However, these studies cannot easily link the identified processes to clinical outcomes, such as MS severity, required for causality assessment of candidate mechanisms. Technological advances now allow the generation of thousands of biomarkers in living human subjects, derived from genes, transcripts, medical images, and proteins or metabolites in biological fluids. These biomarkers can be assembled into computational models of clinical value, provided such models are generalizable. Reproducibility of models increases with the technical rigor of the study design, such as blinding, control implementation, the use of large cohorts that encompass the entire spectrum of disease phenotypes and, most importantly, model validation in independent cohort(s). To facilitate the growth of this important research area, we performed a meta-analysis of publications (*n* = 302) that model MS clinical outcomes extracting effect sizes, while also scoring the technical quality of the study design using predefined criteria. Finally, we generated a Shiny-App-based website that allows dynamic exploration of the data by selective filtering. On average, the published studies fulfilled only one of the seven criteria of study design rigor. Only 15.2% of the studies used any validation strategy, and only 8% used the gold standard of independent cohort validation. Many studies also used small cohorts, e.g., for magnetic resonance imaging (MRI) and blood biomarker predictors, the median sample size was <100 subjects. We observed inverse relationships between reported effect sizes and the number of study design criteria fulfilled, expanding analogous reports from non-MS fields, that studies that fail to limit bias overestimate effect sizes. In conclusion, the presented meta-analysis represents a useful tool for researchers, reviewers, and funders to improve the design of future modeling studies in MS and to easily compare new studies with the published literature. We expect that this will accelerate research in this important area, leading to the development of robust models with proven clinical value.

## Introduction

Multiple sclerosis (MS) is a polygenic, immune-mediated, demyelinating disease of the central nervous system (CNS) that causes substantial personal and societal burden. Understanding the pathophysiology of the initial stages of MS revealed that focal influx of immune cells into CNS tissue can be non-invasively monitored by contrast-enhancing lesions (CELs) on brain magnetic resonance imaging (MRI) (1). CELs, as surrogates of focal inflammation, allowed rapid screening of therapeutic agents (2), identifying many treatments that effectively block the formation of MS lesions.

However, these treatments are not curative, and their efficacy decreases with advancing age at treatment initiation. Indeed, after the age of approximately 54 years, no net benefit on disability progression can be demonstrated in Phase III clinical trials (3). This is partially due to inflammation becoming compartmentalized to CNS tissue during MS evolution (4, 5), making it largely inaccessible to systemically administered treatments. However, neurodegenerative mechanisms (6, 7) likely contribute to the decreasing efficacy of immunomodulatory treatments. To develop effective treatments of MS beyond inhibiting the formation of focal lesions, the MS field must expand its earlier success in gaining pathophysiological insights from early to late disease mechanisms.

Therefore, future therapeutic progress in MS requires the identification and validation of biomarkers that reflect the mechanisms that cause the development of clinical disability in later stages of MS or in patients who no longer form MS lesions thanks to current immunomodulatory treatments. Due to the complexity of these later pathophysiological mechanisms, it is unlikely that a single biomarker can replicate the success of CELs. Indeed, the ability of a single biomarker to reflect key patient-specific outcomes, namely, clinical disability and the rate of its development [as measured by MS severity outcomes (8)] is extremely limited. Consequently, investigators use simple or complex statistical techniques (including machine learning [ML]) to aggregate biomarkers into models with enhanced predictive power.

To our best knowledge, no review exists that summarizes state-of-the-art modeling strategies in MS. The goal of this paper is to present such a critical meta-analysis, to help the MS community, including funders, to identify gaps and opportunities in this important research. We performed a systematic assessment of the technical quality of the reviewed studies, such as sample size, blinding, adjustment for covariates, adjustment for multiple comparisons, integration of healthy volunteer (HV) data to differentiate physiological processes such as aging and gender effects from MS-driven pathologies and, most importantly, we evaluated the level of model validation. Because it has been repeatedly demonstrated that low technical quality (9, 10) and small sample sizes (11–13) overestimate effect sizes and lower the likelihood of reproducible results (14, 15), the attributes we summarize are essential determinants of the generalizability of published models. The broad domain of knowledge included in this work can be utilized as a reference for MS researchers, funders, and reviewers.

## Methods

### Search Method

We conducted a literature search to identify studies that generated statistical models to predict clinical outcomes among patients with MS. This systematic review was conducted in accordance with the Preferred Reporting Items for Systematic Reviews and Meta-Analyses (PRISMA) guidelines. PubMed searches were performed using keywords related to MS, predictive models, and outcomes. Five PubMed searches were performed to identify relevant MRI studies using various combinations of the following keywords: “multiple sclerosis,” “disability,” “correlate,” “MRI,” “machine learning,” “predict,” “AI,” “artificial intelligence,” and “neuroimaging.” Two searches were performed to identify other relevant studies reporting on statistical modeling in MS with the following PubMed search criteria: “[(Multiple Sclerosis [Title/Abstract]) AND (Prediction) AND (Outcome) AND (Model OR Machine Learning)]” on 24 May 2021 and “(((Multiple Sclerosis [Title/Abstract]) AND (Prediction [Title/Abstract]) AND (Outcome))” on 16 August 2021.

### Exclusion Criteria

Two reviewers (JL and EK) independently screened the studies that reported effect sizes for image-, clinical-, or biomarker-based models predicting a clinical outcome. We excluded studies with no predictive models, studies with no imaging, clinical, or biomarker predictors, studies with no clinical outcomes, non-human studies, non-MS studies, and studies with no full text available.

### Information Extraction

The following features were extracted from the methods and results of these studies: (1) types of predictors used for modeling (i.e., clinical, MRI, blood biomarkers, CSF biomarkers, and genes); (2) clinical outcome(s) modeled (e.g., expanded disability status scale (EDSS), secondary-progressive MS (SPMS) conversion); (3) cohort sample size; (4) all reported effect sizes (e.g., for modeling continuous outcomes: *R*^2^ [i.e., coefficient of determination; a statistical measure of how well the regression prediction approximate the measured data], Spearman's ρ [a non-parametric correlation coefficient that measures the strength of association between two variables], Pearson's *R* [a parametric correlation coefficient that measures the strength of association between two variables; should be used only with normally distributed data as it is very sensitive to the effect of outliers]; for dichotomized outcomes such as progression or non-progression: hazard ratios [HR: i.e., an estimate of the ratio of the hazard rate such as disability progression in one vs. other groups: e.g., in treated vs. untreated patients], odds ratios [OR; i.e., the cumulative measure of association between events A and B; with OR = 1 signifying independence between A and B, while OR > 1 signifies that A and B are positively associated while OR <1 means that A and B are negatively associated] and finally, *p*-value [i.e., the probability of obtaining results at least as extreme as observed if the null hypothesis was correct; please note that because *p*-value depends not only on effect size but also on variance and cohort size, it is an extremely poor indicator of effect size alone].

We also extracted seven dichotomized/categorical factors used to assess the quality of the study design (see Section Assessment of the Quality of Study Design in the Reviewed Models). We will refer to these as indicators of the “technical quality” of the study.

### Assessment of the Quality of Study Design in the Reviewed Models

Seven technical quality indicators were extracted from the methods and results sections of each paper, with the following justifications: (1) presence and type of model validation (i.e., (A) independent validation cohort (16) [gold standard] or (B) out-of-bag (OOB)/cross-validation of the training cohort (17). ML algorithms are so powerful that, contrary to expectations, developing models that have surprisingly high effect sizes in the training data set is common and easy. Without a validation strategy, it is not possible to determine the utility of such models, as most artificially and greatly inflate the true effect sizes (15). Thus, the presence and type of model validation are the most important indicators of model reproducibility. The next four attributes of methodological study rigor safeguard against bias. Their pre-specification (e.g., in the protocol) before performing the analysis ensures that the analysis is not modified to increase the likelihood of obtaining the desired results. These include: (2) described process of dealing with outliers to prevent bias (yes/no); useful models should be generally applicable and therefore their effect size should not depend on highly influential observations. Such observations should be identified (and excluded) prior to unblinding by a predefined outlier analysis. If such an analysis is not predefined and the description of methods does not specify how many outliers were excluded and based on what criteria, then the model might be biased (18). (3) Described process of dealing with data missingness (19) to prevent bias (yes/no); another way of modeling results may be biased by excluding observations that do not fit the model post-analysis (e.g., with a justification that these observations were technically inadequate) or by not detecting that some observations were systematically omitted (e.g., measurements were not performed on the sickest patients). Finally, a large amount of missingness that is not disclosed in the paper can also falsely overestimate the generalizability and clinical utility of the model. (4) Adjusting for covariates (yes/no); another way to introduce bias is by failing to detect and adjust for effects of confounding factors that influence the model predictors independently of the outcome (such as age, gender, application of treatments, and different socioeconomic status). For example, such confounders may explain up to 60% of the variance in volumetric brain MRI data (20), which may be mistakenly attributed to the model(s) of neurodegenerative diseases, especially if the patient groups are not carefully matched. (5) Blinding (yes/no); the most effective way to prevent bias during the generation of predictors or during data analysis is to blind the investigators who generate the data, and to perform the aforementioned data cleaning steps before unblinding the data analyst (21). Although randomization is also an essential bias-preventing attribute of methodological design, it is mostly applicable to interventional studies, not to modeling studies. (6) The number of comparisons made (i.e., the number of predictors multiplied by the number of outcomes) and whether *p*-values were adjusted for multiple comparisons (yes/no); this attribute affects the strength of the statistical evidence with which the null hypothesis is rejected. The *p*-value represents the probability of obtaining results at least as extreme as the presented results if the null hypothesis was valid. We would like to present an analogy that provides a reader without statistical knowledge with a practical intuition of how to judge *p*-values in the contexts of performing multiple comparisons: let us imagine we have 20 cards numbered from1 to 20 and we are assessing the ability of a blinded person (i.e., a model) to select the card with the number 1 on it. If this person pulls the card #1 on the first attempt, we may be tempted to conclude that the person knows how to select card #1, as there is only a 5% chance (*p* = 0.05) that she/he will select card #1 on the first attempt randomly. Although we eagerly accept the *p*-value of 0.05 to rule out the null hypothesis in scientific applications related to human health, it is likely that most people would demand stronger evidence that the person can reliably select card #1 in this example. Most people would ask the person to repeat the experiment before they would accept this “model” as valuable. If the person repeats the experiment and selects the card #1 again, then our confidence that she/he knows how to select card #1 will increase to *p* = 0.025 (0.05/2). Now, what happens if the person says that she/he knows how to correctly select the card with a specific number on it: you suggest 19 different numbers and each time the person fails to select the correct one. On the last attempt, you suggest card #1 and the person correctly selects card #1. Will you still conclude that the person represents a good model for selecting card #1? We intuitively understand that if we ignore the previous failed attempts, we reach the wrong conclusion. Yet, when the same is done in reported biomedical research (e.g., the researchers correlated 20 different predictors with the measured outcome and only one of them correlates with *p* = 0.05), we readily accept such a result to reject the null hypothesis. The science of when and how to adjust for multiple comparisons is more complicated (22), but the principle is that we must consider how many comparisons the investigators performed and whether they appropriately adjusted the *p*-values to make a correct inference. (7) Controls utilized (yes/no); this final attribute of the technical rigor deals with the specificity of the model and thus its clinical value: e.g., a model claims to differentiate relapsing-remitting MS (RRMS) from progressive MS. However, when applied to HVs, the model also differentiates two groups of people: younger and older. Clearly, this is not biologically valuable model of MS progression. Or a model claims to be a diagnostic test of MS, buts its accuracy is tested only by differentiating MS from HV, instead of including appropriate controls such as people with non-MS white matter lesions and focal neurological deficits.

Depending on how many of these criteria study fulfilled, the quality of the study design ranged from 0 to 7. Although it is not necessary for a study to fulfill all seven criteria to be reproducible, the score assesses methodological rigor between studies and identifies areas for improvement.

The Master worksheet containing all these extracted data as well as PubMedIDentifiers (PMID) of individual papers is provided as Supplementary Table S1.

### Validation of Published Inverse Relationships Between Study Design Quality and Reported Effect Sizes

Previous studies on non-MS fields showed that (1) small cohort studies; (2) studies of low experimental quality; and (3) studies performed only in the training cohort, all significantly overestimate true effect sizes (10, 11, 13, 14). To assess whether the same can be observed in the MS field we investigated the relationships between the technical quality of studies (including cohort sizes and comparisons of training vs. cross-validation vs. independent validation cohorts) and reported effect sizes.

In addition to univariate analyses, we also classified groups of studies based on the combination of cohort size and technical quality criteria: studies were considered high quality if they reached 1 standard deviation (SD) above the mean for both factors, whereas low quality were 1 SD below the mean for both. To compare all identified low- and high-quality studies (two-sample Wilcoxon [Mann–Whitney] test), we normalized the different metrics of effect sizes to yield common metrics ranging from 0 to 1.

### Public Database Exploration Tool

To allow readers to independently explore the data beyond the relationships described in this paper, we developed a Shiny App in R version 3.6.1. This application includes selection tools that allow user to select all predictor or specific types, all clinical or specific outcomes and all or specific effect size statistic tools and then generates a set of two-dimensional plots that visualize the relationships between the extracted features. The user can also rapidly identify the PMID for a specific study by clicking a specific point in the two-dimensional plots.

## Results

### Clinical Outcomes

**Figure 1:**
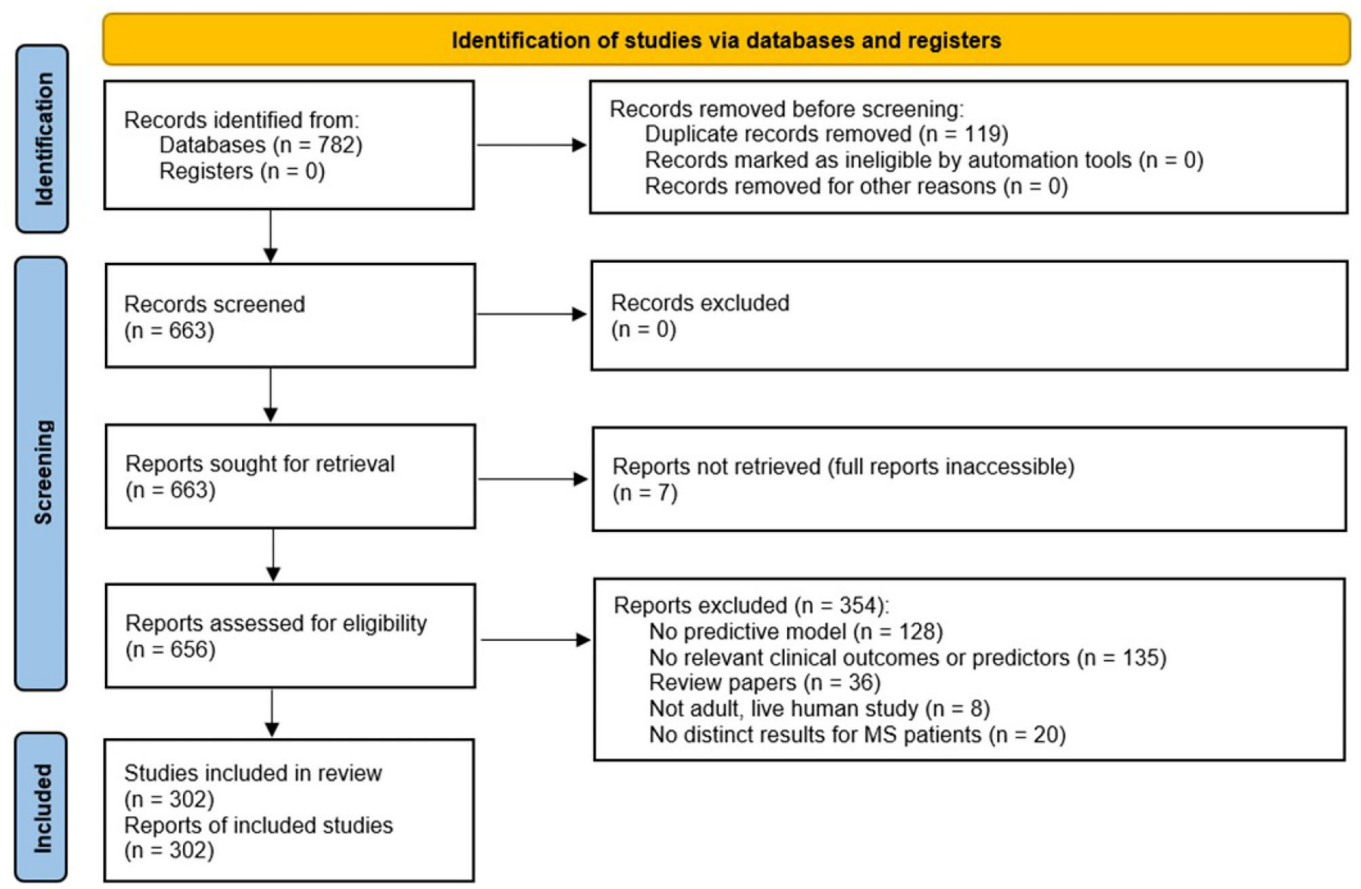
Preferred Reporting Items for Systematic Reviews and Meta-Analyses (PRISMA) chart summarizing the disposition of records identified from PubMed searches. The searches identified 782 records, of which 663 were unique. After several exclusion criteria defined in the figure, 302 unique records were included in the review.

**Figure 2:**
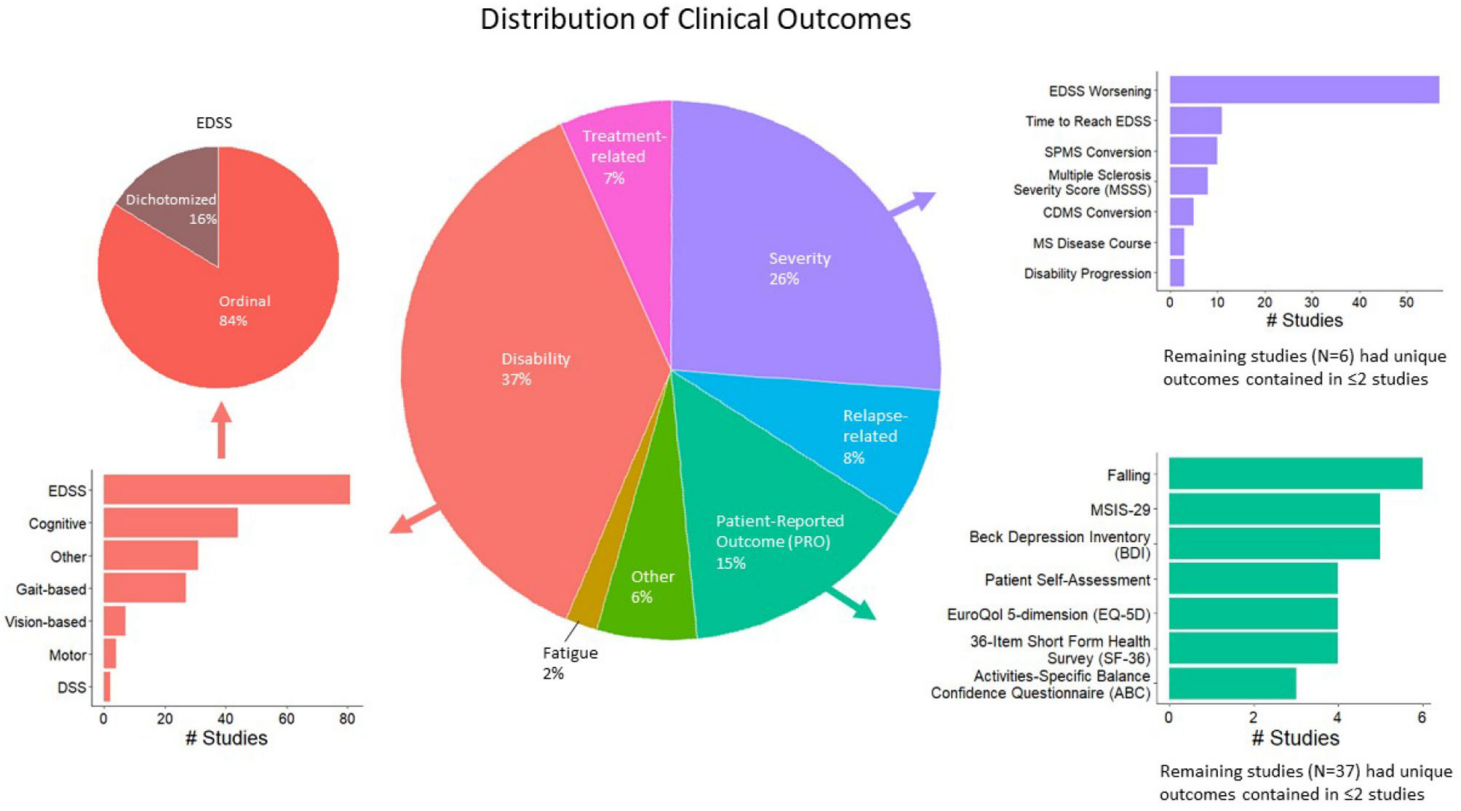
Distribution of modeled clinical outcomes. The large pie chart in the center of the figure shows the distribution of categories of modeled outcomes. The three most frequent outcome categories are disability (37%, red), severity (26%, purple), and patient-reported outcomes (PROs; 15%, teal). The surrounding bar plots show the breakdown of each of these three categories.

A total of 663 studies were screened, excluding duplicate records (Figure 1; PRISMA diagram). After applying the exclusion criteria, 302 studies were included in the review. A total of 189 clinical outcomes were predicted in the 302 included studies. The breakdown of outcomes by category is shown in Figure 2.

The largest category of clinical outcomes was *MS progression as measured by traditional disability outcomes* (Figure 2, red color; 37% of the studies reviewed). Of these, the most prevalent outcomes were EDSS-based (*n* = 81 studies), such as predicting EDSS on an ordinal scale, followed by the prediction of EDSS as a dichotomous variable. Cognitive disability outcomes constituted the second largest subcategory (*n* = 44). These included the Paced Auditory Serial Addition Test (PASAT), the Stroop test, the Symbol Digit Modalities Test (SDMT), etc. The third most prevalent progression outcomes were gait-based (*n* = 27), which included the timed 25-foot walk (T25FW), Hauser ambulation index, 6-min walk test, Timed Up and Go [TUG], dynamic gait index, etc.

Following MS progression/disability outcomes, the next largest category of outcomes was *MS severity outcomes*, which were modeled by 26% of the studies reviewed (Figure 2, purple color). A total of 69 studies predicted changes in EDSS over time, including EDSS worsening and time to reach a specific EDSS score. A total of 10 studies predicted the conversion to SPMS, eight predicted EDSS-based MS Severity Score (MSSS), five predicted conversions to clinically definite MS, and the remaining outcomes were studied by fewer than five studies.

Finally, *patient-reported outcomes* (PROs; Figure 2, teal color) were modeled by 15% of the studies reviewed. This category was fractionated, with falls predicted in six studies, the MS Impact Scale (MSIS-29) and Beck Depression Inventory (BDI) by five studies each. The remaining outcomes were studied by fewer than five studies.

### Predictor Variables

**Figure 3:**
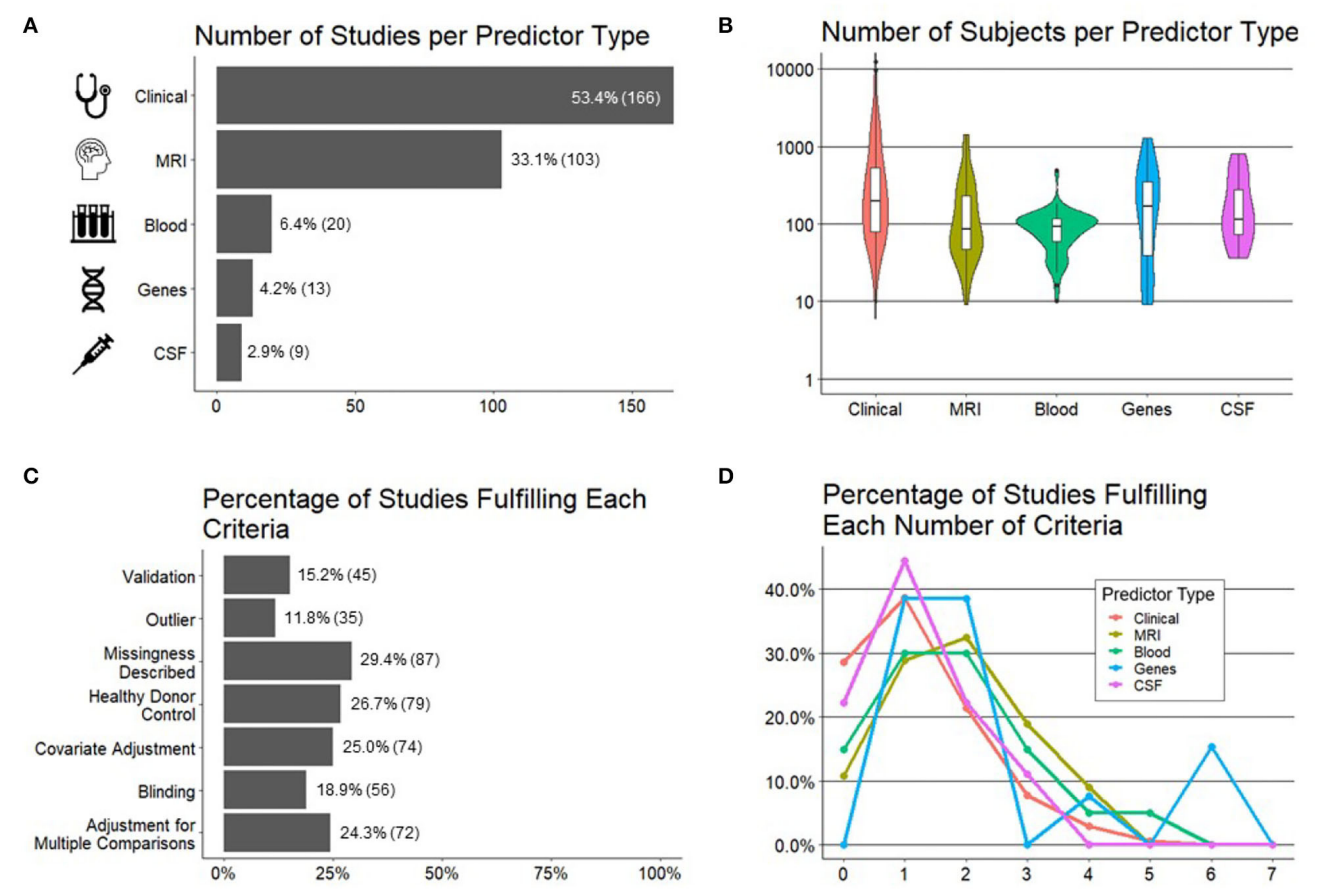
Important characteristics of the studies reviewed. **(A)** Number of studies (*x*-axis) per predictor type (*y*-axis). **(B)** Number of subjects (*y*-axis) by predictor type (*x*-axis). **(C)** Percentage of studies (*x*-axis; number of studies in parentheses) fulfilling each preselected criteria of experimental design/technical quality of the study (*y*-axis). **(D)** Percentage of studies (*y*-axis) per each predictor type fulfilling a number of technical criteria (*x*-axis).

Five categories of predictor variables were used in these models, namely, clinical (*n* = 166 studies), MRI (*n* = 103), genes (*n* = 13), blood biomarkers (*n* = 20), and CSF biomarkers (*n* = 9) (Figure 3A).

We hypothesized, and confirmed, that the sample sizes would be the largest for models using clinical predictors because they are the easiest to collect. Using similar reasoning, we expected the smallest sample sizes for CSF predictors due to an invasive nature of lumbar punctures. Instead, we observed the smallest sample sizes for models utilizing MRI predictors and blood biomarkers, where most studies had sample sizes of <100 patients, with some as low as 10 patients (Figure 3B).

### Technical Quality

In addition to recording cohort sizes for each study reviewed, we collected seven study design factors aimed to minimize bias (see Section Methods for details) and therefore maximize the probability that the reported results would be generalizable (Figure 3C). These were: (1) blinded analyses; (2) pre-defined/described missing data; (3) pre-defined/described methodology for outlier identification and removal to minimize bias; (4) adjustment for covariates; (5) presence of controls, such as HVs, to differentiate physiological processes, such as aging or gender effects, from MS-related processes; (6) the number of comparisons performed and whether investigators employed any strategy to adjust significance thresholds if the number of comparisons was high; and finally (7) the level of model validation (if any), differentiating cross-validation methods that reuse training cohort samples from true independent cohort validation, considered the gold standard.

Although no study needs to fulfill all seven criteria to yield reliable results, it was unexpected to observe that majority of the studies fulfilled one or fewer criteria and only 1% of the studies fulfilled more than four. When comparing the technical quality of studies based on different predictors (Figure 3D), we observed the highest technical quality of the studies that used genes, followed by MRIs and blood biomarkers. Astonishingly, more than 20% of the studies that used clinical or CSF biomarker predictors fulfilled zero technical quality criteria.

Finally, because current modeling algorithms are highly susceptible to overfitting, an essential determinant of model's generalizability is the level of its validation. Overfitting is caused by the ability of ML algorithms to find and amplify subtle changes in the data, including noise, to achieve fit that is much stronger than biologically plausible. Consequently, when the model is applied to a new set of samples/patients, it will have a much lower fit or may not validate at all. There are two types of validation: the first reuses training cohort data, in various manners that are beyond the scope of this review. It is often called “cross-validation” or “OOB data.” We will use term “cross-validation” to signify any validation strategy that reuses training cohort data. To what degree cross-validation faithfully predicts the generalizability of the model depends on the details of how it was performed. Cross-validation may be overly optimistic if researchers fail to prevent bias, and this is often the case. Therefore, the gold standard is independent cohort validation, which implies using the model on a new set of samples/subjects that did not contribute, in any way, to model generation.

We observed that only 15% of the studies used any type of validation with only 8% of all studies used independent validation.

### Effect Sizes

Effect sizes for each of these studies were included as reported (for an explanation of these metrics, see Section Methods). The most reported metric was *R*^2^ in 101 studies with Pearson's *R* being reported in 53 studies, HR in 46 studies, OR in 43 studies, and Spearman's ρ in 29 studies. Values of *p* were reported alongside these metrics in 202/302 studies.

Overall, we observed a highly selective, rather than comprehensive use of statistical outcomes that reflect effect sizes. This selectivity limits the ability to compare effect sizes between different studies.

### Association Between Study Quality and Effect Size

It is estimated that between 51% and 89% of the published literature in biomedical sciences is not reproducible (15, 23, 24) and poor study design, based on small sample sizes (11, 13) and the failure to prevent bias (25–27) is the major contributor to this reproducibility crisis. Indeed, as outlined in the introduction, previous studies highlighted an inverse relationship between the technical quality of study design (10) [including cohort sizes (11, 13)] and reported effect sizes, validating the notion that the technical quality of study design is a major determinant of the generalizability of gained scientific knowledge.

**Figure 4:**
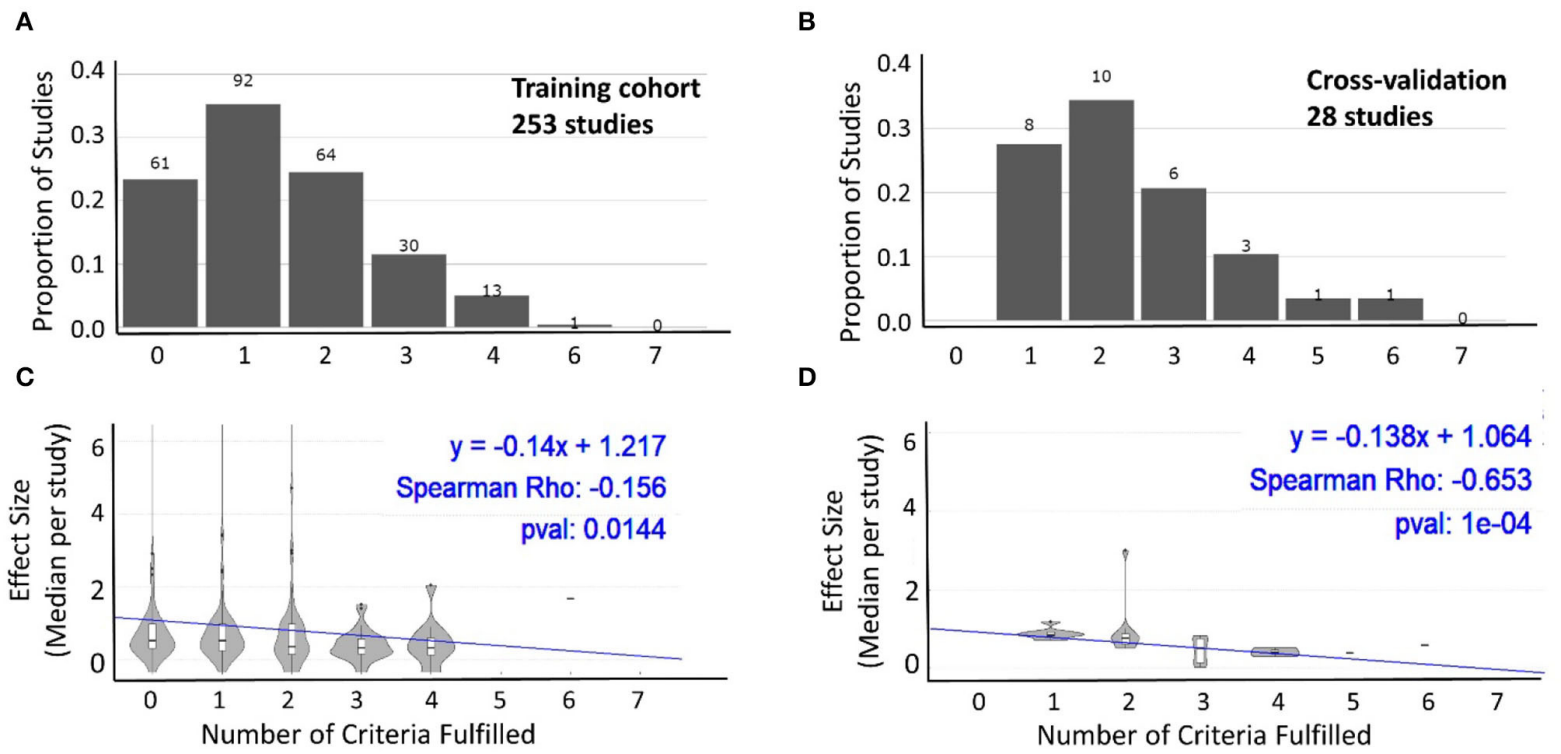
Relationship between technical quality of the study and reported effect size. The proportion of studies fulfilling the sum of the seven technical quality criteria (zero weakest experimental design to seven strongest experimental design) for 253 studies with training cohorts **(A)** and cross-validation cohorts **(B)**. The number of studies in each category is listed above the bars. Effect sizes reported by studies categorized based on the number of technical quality criteria they fulfilled (0–7) for training cohort (**C**; *n* = 253) and cross-validation (**D**; *n* = 28) results. In both cohorts, the reported effect sizes decreased as the number of technical quality criteria fulfilled by these studies increased.

To assess whether we can identify analogous inverse relationships between reported effect sizes and our pre-defined systematic grading of technical quality of the reported study design, we performed two types of analyses. In first analysis, we compiled all studies that reported any effect size separately for the training (Figure 4A) and cross-validation cohorts (Figure 4B). We then assessed whether there is any relationship between the number of technical quality criteria a study fulfilled vs. the reported effect size. For both the training cohort data (Figure 4C) and cross-validation (Figure 4D), we observed an inverse relationship between the technical quality of the study and the reported effect size.

**Figure 5:**
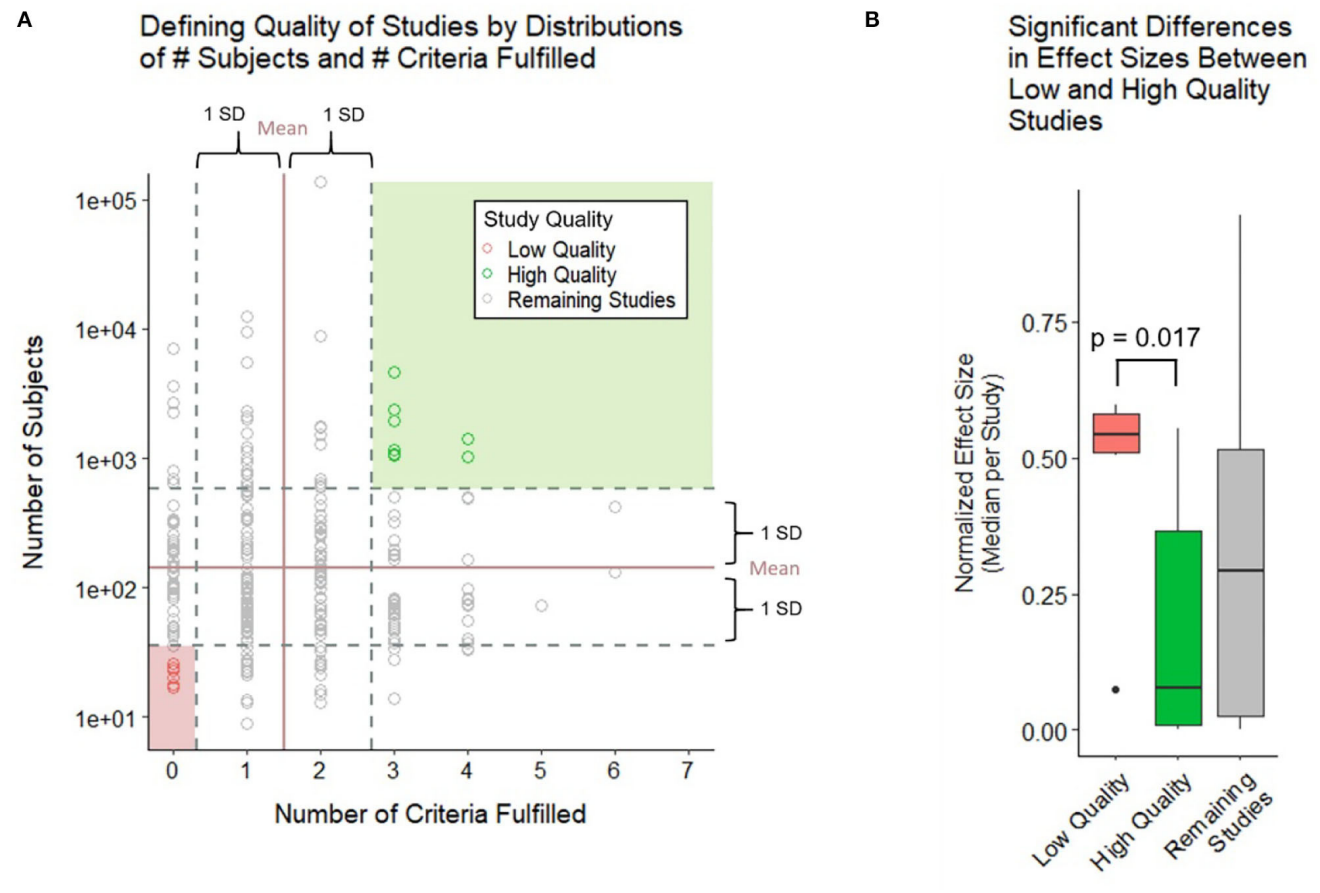
Relationship between technical quality and sample size of the study and reported effect sizes. **(A)** Study quality is defined by the number of subjects and number of criteria fulfilled with high quality studies falling 1 SD above both criteria and low-quality studies falling 1 SD below both criteria. **(B)** Boxplot compares the normalized effect sizes between low- and high-quality studies using a two-sample Wilcoxon (Mann–Whitney) test. Low-quality studies were found to have higher effect sizes at a significant *p*-value of 0.017.

Because the above strategy ignored cohort size, which is an important determinant of model generalizability, in the second analysis we construed the two-dimensional assessment of the study design (Figure 5A), integrating both grading of reported technical quality with reported sample sizes. Using means ± one SD of all studies, we identified low-quality studies (i.e., at least one SD below the average for both technical quality and sample size) vs. high-quality studies (i.e., at least one SD above the average for both domains). We observed significantly higher reported standardized effect sizes for low quality studies compared with high quality studies (Figure 5B). As expected, effect sizes for the remaining studies were centered between the low- and high-quality studies.

### Effect Sizes for EDSS-Based Models of MS Progression and MS Severity

To facilitate the interpretation of any future models, we compared the strength of models for different predictors using EDSS-based MS progression (Table 1) and MS severity outcomes [MSSS and age-related MSS (ARMSS); Table 2]. EDSS-based outcomes are the most broadly used in MS field. We found them to be modeled most, and they are accepted by regulatory agencies for assessing the therapeutic efficacy of MS drugs. For each outcome and predictor pair, we provide the highest reported effect size and the effect size reported by the study of highest technical quality. Whenever available, we also reported effect sizes for cross-validation and independent validation studies.

**Table 1:** This set of tables shows the models of expanded disability status scale (EDSS; modeled as ordinal scale) using the following predictor types: clinical, magnetic resonance imaging (MRI), and blood, respectively.

**Cohort**	**Study type**	**R^**∧**^2 (PMID, #QC, *N*)**	**|Spearman ρ| (PMID, #QC, *N*)**	**|Pearson R| (PMID, #QC, *N*)**
**Outcome: EDSS**	**Predictor: Clinical**
Training	Strongest effect size	0.67 (31218917, 1, 100)	0.77 (18184917, 0, 161)	0.51 (32615409, 1, 38)
	Highest quality	0.26 (26362898, 2, 362)	0.61 (31218917, 1, 100)	-
Cross-validation	Strongest effect size	-	-	-
	Highest quality	-	-	-
Independent validation	Strongest effect size	-	-	-
	Highest quality	-	-	-
**Outcome: EDSS**	**Predictor: MRI**			
Training	Strongest effect size	0.64 (33598931, 1, 115)	0.82 (24508617, 1, 9)	0.36 (20373349, 0, 107)
	Highest quality	0.52 (30657011, 3, 366)	0.49 (18556361, 4, 74)	0.26 (26115736, 3, 195)
Cross-validation	Strongest effect size	0.19 (32924846, 2, 250)	-	-
	Highest quality	-	-	-
Independent validation	Strongest effect Size	-	-	-
	Highest quality	-	-	-
**Outcome: EDSS**	**Predictor: Blood**			
Training	Strongest effect size	0.19 (31801106, 1, 23)	-	0.47 (31801106, 1, 23)
	Highest quality	0.06 (30564615, 3, 117)	-	0.15 (22354743, 2, 68)
Cross-validation	Strongest effect size	-	-	-
	Highest quality	-	-	-
Independent validation	Strongest effect size	-	-	-
	Highest quality	-	-	-

*Effect sizes were reported for studies with the strongest effect sizes as well as for studies with the highest quality. The following metrics were explored: R^2^, Spearman's ρ, and Pearson's R. PMID, PubMed unique Identifier; #QC, sum of technical quality criteria that the study fulfilled; n, number of subjects in the study*.

**Table 2:** This set of tables includes EDSS-based multiple sclerosis (MS) severity outcomes MS Severity Score (MSSS) and age-related MSS (ARMSS), showing the studies that reported the highest effect sizes and those that achieved the highest technical quality, reporting *R*^2^, Spearman's ρ, and Pearson's *R*.

**Cohort**	**Study type**	**R^**∧**^2 (PMID, #QC, *N*)**	**|Spearman ρ| (PMID, #QC, *N*)**	**|Pearson *R*| (PMID, #QC, *N*)**
**Outcome: MSSS**	**Predictor: MRI**
Training	Strongest effect size	0.45 (24122185, 1, 67)	-	-
	Highest quality	-	-	-
Cross-validation	Strongest effect size	-	-	-
	Highest quality	-	-	-
Independent validation	Strongest effect size	-	-	-
	Highest quality	-	-	-
**Outcome: MSSS**	**Predictor: Blood**
Training	Strongest effect size	0.24 (20965962, 2, 54)	-	0.19 (22354743, 2, 68)
	Highest quality	-	-	-
Cross-validation	Strongest effect size	-	-	-
	Highest quality	-	-	-
Independent validation	Strongest effect size	-	-	-
	Highest quality	-	-	-
**Outcome: MSSS**	**Predictor: Genes**
Training	Strongest effect size	0.16 (20378664, 2, 605)	-	-
	Highest quality	-	-	-
Cross-validation	Strongest effect size	-	-	0.58 (31396954, 6, 205)
	Highest quality	-	-	-
Independent validation	Strongest effect size	-	0.06 (31396954, 6, 94)	0.20 (31396954, 6, 94)
	Highest quality	-	-	-
**Outcome: ARMSS**	**Predictor: Genes**			
Training	Strongest effect size	-	-	-
	Highest quality	-	-	-
Cross-validation	Strongest effect size	-	-	0.58 (31396954, 6, 205)
	Highest quality	-	-	-
Independent validation	Strongest effect size	-	0.12 (31396954, 6, 94)	0.17 (31396954, 6, 94)
	Highest quality	-	-	-

*PMID, PubMed unique Identifier; #QC, sum of technical quality criteria that the study fulfilled; n, number of subjects in the study*.

For modeling MS progression using the ordinal EDSS (Table 1), we found comparable highest reported effect sizes between studies that used clinical (i.e., *R*^2^ = 0.67) and MRI (*R*^2^ = 0.64) predictors. The decrease in effect size for best-in class studies was larger for clinical predictors (i.e., *R*^2^ = 0.26) than for MRI predictors (*R*^2^ = 0.52). Only MRI predictors reported cross-validation results, which further decreased the effect size to *R*^2^ = 0.19. We identified no independent validation cohorts. Blood biomarker predictors achieved a much lower effect size in predicting EDSS: the strongest effect size (*R*^2^ = 0.19) was reported by a study that included only 23 subjects and achieved the technical quality score of 1, whereas the highest quality study reported *R*^2^ = 0.06. We identified no cross-validation or independent validation studies for blood predictors of EDSS. Finally, we identified no studies reporting genetic or CSF biomarker-based predictors of EDSS.

For predicting MS severity (Table 2) measured by MSSS, the strongest reported effect size was *R*^2^ = 0.45 for MRI and *R*^2^ = 0.24 for clinical predictors. However, these were derived from small training cohorts (*n* = 67 for MRI and *n* = 54 for clinical predictors) and were not validated. We identified several studies using genetic predictors of MS severity; effect sizes for independent validation of MSSS and ARMSS were reported only as correlation coefficients and ranged from Pearson's *R* = 0.17–0.2. We did not identify any blood or CSF biomarker-based models of MS severity.

### Shiny-App Exploration Tool

To facilitate independent exploration of the rich data set we collected beyond the Excel worksheet containing all extracted data and deposited as Supplementary Table S1, we also developed the Shiny App that allows selective filtering of the data (e.g., to isolate specific predictors, specific outcomes, and specific statistical metrics of effect sizes). It can be found at the following link: https://jliu159.shinyapps.io/MS_Models_LitSearch_Data_Exploration/. This tool was designed to facilitate comparisons of any future models with the reviewed literature. A user manual can be found in the Supplementary Material.

## Discussion

Technological advances make measuring thousands of genes, transcripts, proteins, and metabolites and hundreds of imaging and clinical biomarkers relatively easy and common. Thanks to analogous computational advances, these measurements can be aggregated into models that are expected to elucidate disease mechanisms and provide clinical (e.g., prognostic) value. These are valuable developments; however, to fulfill the expectations of providing reproducible knowledge and clinical value, these technological advances must be paired with the rigor of experimental design.

This review shows great potential to improve modeling of clinical disease characteristics in MS. It is startling that 21% of the published studies failed to implement *any* of the seven attributes of a strong experimental design (9, 11–13, 15) to limit bias and enhance reproducibility. An additional 36% of the studies reviewed implemented only one of the seven technical criteria, making this the median attribute of experimental design quality in MS models. This is clearly suboptimal.

This inferior experimental design is compounded by the frequent use of small sample sizes (i.e., fewer than 100 subjects): in fact, for MRI and blood non-genetic biomarker studies, the median cohort sizes were <100. Considering the complexity of disease mechanisms in polygenic diseases like MS, a modeling cohort of <100 patients with MS cannot comprise the entire spectrum of disease heterogeneity. Moreover, such small studies are highly susceptible to bias (11, 13), especially when <20% used blinding, <25% adjusted for covariates, and <30% addressed missingness or adjusted the threshold of significance for the number of comparisons performed (sometimes more than hundreds).

Evidence from other scientific areas (10, 11, 13, 14), supported by this paper, shows that poor experimental design, intensified by small cohort sizes, overestimates effect sizes. This is inevitable, as statistical power is positively associated with cohort and effect sizes (25). Consequently, the only way for small studies to reach statistical significance is for them to demonstrate unusually high effect sizes. These high effect sizes are almost always inflated as abnormalities in individual transcripts, proteins, or metabolites are only mild or moderate, with severe disturbances being incompatible with life (28).

Another underappreciated aspect of complex modeling algorithms is their incredible overfitting power. Contrary to laymen's understanding, it is surprisingly easy to derive seemingly strong models in training cohorts, especially if one measures a comparably higher number of biomarkers to the number of subjects. Such disproportional richness of predictors poses a high probability of spurious associations between predictors and the outcome(s), akin to the example we introduced in Section Methods when explaining the ease of making the wrong conclusion if we fail to consider how many “comparisons” were performed during the modeling strategy. Thus, the validation of such models is essential: the probability that the same spurious (i.e., not caused by biology) relationship(s) will occur again in the completely independent set of observations is low. However, validation was included only in 15% of all studies, and most of these (56%) used cross-validation rather than independent validation. Indeed, <8% of all studies validated their model(s) on a completely new set of subjects (i.e., independent validation cohort), which is the gold standard.

Cross-validation (also called rotation estimation or OOB testing) reuses some of the training cohort data by partitioning or resampling the data to train and test models on different iterations. For example, a training cohort may be randomly partitioned (many times) to generate “internal” training and validation splits; this partitioning may be as large as 50:50 split or as small as leaving out only one sample. The model then tests the accuracy of the predictions of these OOB samples. Because cross-validation does not require any new data sets, it should be included in all studies, not just 10% of them. Although cross-validation is certainly better than no validation, it may still overestimate the power/accuracy of the classifier in comparison to true independent validation (29). We have *always* observed decreases in model performance (e.g., predictive accuracy) from training cohort to cross-validation and from cross-validation to independent validation (30–32). These decreases happen regardless of whether we use clinical data (33), functional data (34), MRI data (32, 35), soluble biomarkers (30, 36), or genes (31); and they are often substantial, especially when comparing cross-validation with true independent validation [e.g., from *R*^2^ 0.72 in the training cohort to 0.64 in the 5-fold cross-validation with 10 repetitions to 0.01 in the independent validation (34)]. Please note that the effect sizes for the EDSS-based outcomes summarized in Tables 1, 2 also show decreasing effect sizes with increasing quality of experimental design, and from training to cross-validation results. Finally, we emphasize that an exceptionally low *p*-value achieved in the training cohort (even in the cross-validation cohort) does not guarantee the dramatic loss of model accuracy observed in the independent validation cohort (15, 31).

Cross-validation frequently overestimates the accuracy of the model because it often includes a circular argument: somewhere in the modeling process the OOB samples contributed to model construction. For example, we already mentioned that “overfitting” tends to happen when models are generated from a disproportionally large number of predictors in comparison to the number of observations. To avoid this problem, the data analyst may “constrict” the number of predictors for model development, e.g., by correlating predictors with the modeling outcome and selecting only predictors with significant correlations. If this initial step was done in all training cohort observations (which is usually the case), the OOB samples were “compromised”; they contributed to model development and, therefore, will likely overestimate the model effect size in comparison to independent validation.

Furthermore, these early modeling steps (such as quality control, outlier removal, and feature selection), if performed unblinded, may introduce bias and are often omitted from the publication altogether [a problem called “selective reporting” (37–39)]. Consequently, bias may not be identified during the review process. Another source of bias that leads to major misinformation in the scientific literature is publication bias (40): when so-called “positive” studies (i.e., those that achieved arbitrary the value of *p* < 0.05) are published, but “negative” studies, including negative independent validation studies, frequently remain unpublished. This collectively causes unrealistically optimistic view of the reproducibility of the published results.

We initiated this work with the goal of identifying opportunities to advance the modeling of MS outcomes. Based on this work, we endorse the following recommendations:

### Enhance the Experimental Design of Future Studies

To minimize bias and maximize reproducibility, no modeling study should fulfill less than four criteria of sound experimental design, and all should include at minimum cross-validation. Studies should also be of sufficient size, including all MS phenotypes, to increase the probability that the results will be generalizable.

### Include Most Common Outcomes (E.g., EDSS-Based) as Comparators

Although modeling new and possibly better clinical or functional outcomes (including PROs) are desirable, unless EDSS-based outcomes are included, it is impossible to compare different models and understand their clinical utility.

### Prioritize Modeling Continuous (or Ordinal) Over Dichotomized Outcomes

Even though the EDSS is an ordinal scale and EDSS-based severity outcomes (i.e., MSSS and ARMSS) are continuous, 71/138 (51%) studies used the EDSS in a dichotomized manner: e.g., predicting progression (yes/no) within a certain period. Of the 71 studies that used dichotomized EDSS-based outcomes, dichotomization was not uniform across studies. For example, EDSS worsening was defined as a 1-point increase in one study, a 0.5-point increase in another study, and a 0.5- or 1-point increase depending on some EDSS threshold, which varied between EDSS 4 and 6. Without justification for a specific definition of EDSS dichotomization and assurance that this definition was selected before data analyses, non-uniform selection of EDSS-based outcomes may lead to bias, while also preventing comparison between studies. Such call for greater standardization of clinical outcomes has been made previously in the MS field (16). We strongly recommend that even studies that chose to dichotomize the EDSS-based outcome include models that predict the EDSS as an ordinal scale and MSSS/ARMSS as continuous scales. Predicting when and how much progression will occur is a mathematically harder problem than predicting whether a patient is likely to progress. While the dichotomized model may predict that two patients will progress in the next 5 years, the continuous model may predict that one patient will progress 3 EDSS points starting next year and another will progress 0.5 EDSS points by the 5th year. This level of granularity, if validated, provides a greater biological insight into the mechanisms of disease progression and a stronger information gain for clinical management. Because the data (i e., EDSS) are already collected, applying different modeling strategies and reporting their outcomes are not difficult.

### Report Broad and Accurate Metrics of Model Accuracy

We observed highly inadequate reporting of model accuracy metrics, at times limited only to the p-value. Values of p do not reliably reflect model accuracy; in fact, one can get a low p-value for a model that has an inverse relationship with a measured outcome. Or, in large cohorts, a clinically insignificant model (explaining <1% of the variance) may have a surprisingly low p-value. For continuous outcomes, correlation coefficients only reflect the strength of the association between measured and predicted outcomes, but not the accuracy of the model: e.g., let us imagine that measured and predicted outcomes are distributed in perfect (positive) line, resulting in correlation coefficients of 1. However, while the measured EDSS has spread of values between 0 and 10, the predicted EDSS may have a different spread of values: e.g., 4–6 or 1–2. In fact, such “mis-calibrated” models are quite common. The R2, reflecting the proportion of the variance explained by the model is preferable to correlation coefficients. However, the best indicator of model accuracy reflects how closely the model predictions match the absolute values of the measured outcomes (i.e., 1:1 line), such as Lin's concordance coefficient (CCC). Current statistical packages, including freely available options such as R, can calculate all these statistical parameters. Their reporting will provide a better assessment of model accuracy and would facilitate comparison between studies.

### Addressing the Clinical Utility of the Models

Not all models have, or must have clinical utility; as indicated above, molecular, genetic, or cellular biomarker predictors might be useful by simply linking specific pathophysiological processes or pathways to MS clinical outcomes. However, even these models should assess and publish metrics of clinical utility, such as receiver operating characteristic (ROC), accuracy, sensitivity/specificity, and positive and negative predictive values, so that clinicians correctly understand their potential clinical value (or lack thereof).

### Validation of the Most Promising Observations in the Independent Cohort(s)

The low rate of independent validation (i.e., 8% of the studies) observed in this meta-analysis is, unfortunately, consistent with similar reports of very low independent validation rates (17). Because a “lack of validated predictive tools in MS” has been recognized before (18), funders need to devote more funding to high-quality, definite independent validation studies. Analogously, reviewers and readers should recognize that training cohort data, even cross-validation, has high probability to overestimate the generalizability of the model(s), and reward publications that include independent validation cohorts.

### Deposit the Raw Data

Most journals do not limit the amount of Supplementary Data. Data sharing is essential to independently validate the algorithms that underlie published models, but also to explore stronger algorithms/models.

## Conclusions

Finally, as evidenced by the summary of current EDSS-based models, we identified a strong need to develop validated models of MS clinical outcomes using cellular or molecular biomarkers. Vast majority of the models reviewed used clinical or MRI predictors. Although they may provide clinical value, they are less likely to yield the mechanistic insight into MS progression or MS severity necessary for the development of effective treatments for progressive MS or treatments that would abrogate the accumulation of disability in patients treated by current disease-modifying agents that successfully limit the formation of new lesions.

While most of these recommendations have no financial or logistical implications (i.e., they can be performed immediately on existing cohorts as they relate to the analytical steps of model development), increasing cohort sizes, and especially the inclusion of independent validation cohorts, requires substantial financial and human resources and cannot be accomplished without funders recognizing the importance of such properly powered studies and prioritizing them for financial support.

## Data Availability Statement

The original contributions presented in the study are included in the article/Supplementary Material, further inquiries can be directed to the corresponding author.

## Author Contributions

JL and EK performed the literature search and extracted all data for this meta-analysis. JL analyzed the data, generated the figures and Shiny App, and contributed to this paper. BB construed the project conceptually, guided and supervised all aspects of this study, and contributed to the writing of this paper. All authors critically reviewed and edited this paper.

## Funding

This study was supported by the intramural research program (IRP) of the National Institute of Allergy and Infectious Diseases (NIAID) at the National Institutes of Health (NIH).

## Conflict of Interest

The authors declare that the research was conducted in the absence of any commercial or financial relationships that could be construed as a potential conflict of interest.

## Publisher's Note

All claims expressed in this article are solely those of the authors and do not necessarily represent those of their affiliated organizations, or those of the publisher, the editors and the reviewers. Any product that may be evaluated in this article, or claim that may be made by its manufacturer, is not guaranteed or endorsed by the publisher.
